# The Mineral Apposition Rate on Implants with Either a Sandblasted Acid-Etched Implant Surface (SLA) or a Nanostructured Calcium-Incorporated Surface (XPEED^®^): A Histological Split-Mouth, Randomized Case/Control Human Study

**DOI:** 10.3390/ma17133341

**Published:** 2024-07-05

**Authors:** Abdallah Menhall, Pierre Lahoud, Kyung Ran Yang, Kwang Bum Park, Dainius Razukevicius, Tonino Traini, Christian Makary

**Affiliations:** 1 Oral Surgery Department, Saint Joseph University of Beirut, Beirut 1104 2020, Lebanon; abdallah.menhall@usj.edu.lb (A.M.); pierre.lahoud@usj.edu.lb (P.L.); christian.makary@usj.edu.lb (C.M.); 2 Daegu Mir Dental Hospital, Jung-gu, Daegu 41934, Republic of Korea; ddsykr@gmail.com; 3 MegaGen Implant Co., Ltd., Daegu 42921, Republic of Korea; periopkb@imegagen.com; 4 Faculty of Odontology, Lithuanian University of Health Sciences, 44307 Kaunas, Lithuania; dainius.razukevicius@lsmu.lt; 5 Department of Innovative Technologies in Medicine & Dentistry, University “G. d’Annunzio” of Chieti Pescara, 66100 Chieti, Italy

**Keywords:** implantology, osseointegration, bone turnover, surface treatment, nanostructured calcium-incorporated surface, tetracycline bone labeling, mineral apposition rate

## Abstract

This study aimed to histologically evaluate the effects of XPEED^®^ and SLA surface on the mineral apposition rate (MAR) at 3 and 5 weeks in titanium dental implants placed in human bone. In total, 17 titanium dental implants with XPEED^®^ surface (*n* = 9) used as test and SLA surface (*n* = 8) used as control were included in this study. Each patient received four doses of tetracycline 500 mg at 12 h intervals 2 weeks prior to biopsy retrieval. Implant retrieval was performed, and retrieved biopsies were carefully treated for histomorphometric evaluation under epifluorescence microscopy. At 3 and 5 weeks, newly formed bone appeared in direct contact with both types of tested surfaces. At 3 weeks, the MAR value was, respectively, 2.0 (±0.18) μm/day for XPEED^®^ implants and 1.5 (±0.10) μm/day for SLA implants (*p* = 0.017). At 5 weeks, lower MAR values for both XPEED^®^ and SLA implants were noted, with 1.2 (±0.10) μm/day and 1.1 (±0.10) μm/day, respectively (*p* = 0.046). The overall evaluation by linear regression analysis for both time and implant surfaces showed a decreased osteoblast activity at 5 weeks compared to 3 weeks (*p* < 0.005). The results of the present study show that the bone apposition rate occurs faster around implants with XPEED^®^ surface at 3 weeks and 5 weeks of healing. MAR values may support the use of implants with XPEED^®^ surfaces in early loading protocols.

## 1. Introduction

Dental implant placement triggers a series of biological events in the surrounding bone, a healing process similar to that of bone injury, where there is new, immature bone apposition between the implant surface and pristine residual bone, leading to new bone formation and apposition on fixture surface, provided there are no major complications, which will ultimately result in osseointegration [1]. The speed, rate, and reliability of this process are among the main research subjects in implantology today and would ultimately determine the efficiency of a given implant type [2]. This phenomenon depends on many variables mainly involving patient, clinician, and materials. One of the major elements in this equation is implant surface topography, which can be a decisive factor in treatment duration, protocol, and overall success [3,4]. Modern implantology largely relies on surface performance, especially in immediate and early implant placement and loading, as well as in maintaining safe and healthy peri-implant bone levels [5]. Recently, many advances have been achieved in that area, allowing for more reliable and predictable treatments as well as shortened healing periods [6]. Modern roughened implants yield noticeably superior performance when compared to their machined predecessors, with increased bone fixation and bone-to-implant contact [7]. Sandblasted, large-grit acid-etched surfaces have been the subject of many publications and have shown very good performance, be it in early bone apposition or its long-term stability [8]. Recent studies have shown very high success rates and tissue stability at 10-year follow-up periods for both immediate and early-loaded implants with SLA surfaces [9]. In a recent publication histologically comparing implants with three types of surface (SLA, machined, and nanostructured calcium-incorporated surface (XPEED^®^)) retrieved from human patients 4 to 6 weeks after placement, values for XPEED^®^ at 4 weeks were significantly higher [10]. However, the study showed no significant difference between SLA and XPEED^®^ values at 6 weeks, allowing the authors to conclude that nanostructured calcium titanate coating could enhance peri-implant bone deposition at early healing stages and further reduce loading periods to as low as 4 weeks. The bone apposition rate is a function of bone matrix synthesis and initial mineralization, and it is indicative of osteoblast cell activity [11]. Mineral apposition rate (MAR) measurement is an efficient diagnostic tool for classifying and evaluating the progression of various metabolic bone diseases [12]. In implant dentistry, it is valuable in obtaining a clear image of bone behavior around various implanted materials [13]. It allows for the measurement of the distance between bone labels over time at the implant–bone interface region, for a deeper understanding of peri-implant bone healing [14]. MAR measurement is sometimes used in conjunction with fluorochrome bone labeling for observing bone formation dynamics at selected time points, which also provides objective insight for differentiating necrotic and non-necrotic bone for diagnostic and therapeutic purposes [15]. In this type of labeling, tetracyclines are widely used for their variety and distinctive yellow/green emission spectra and have shown to be well tolerated by patients when administered for this purpose [16]. Because of their affinity to calcium, tetracycline and other fluorochromes have been shown to become incorporated into bone after entering the bloodstream, mainly in areas of bone remodeling and bone apposition, given their affinity for new mineralized tissue formation sites where they bind to bone mineral through calcium ion chelation at the surface of newly formed apatite crystals [17]. Bone labeling allows for the relatively clear and objective visualization of bone modeling and remodeling under a fluorescent microscope [18]. It has been effectively used for the quantitative measurement of bone formation and remodeling dynamics, comparing the osteointegration environment and assessing the osteogenesis rate and proximity to the implant surface, as well as the mineral apposition rate and osseointegration index values [19]. The main objective of the present study was to compare, at 3 and 5 weeks, MAR in implants having either sandblasted, large grit, acid-etched implant surface (SLA) or a nanostructured calcium-incorporated surface (XPEED^®^), in patients prescribed oral tetracyclines 2 weeks prior to biopsy retrieval. The null hypothesis (H_0_) under study considered no statistically significant differences in MAR between SLA and XPEED^®^ surfaces at both of the early healing times considered.

## 2. Materials and Methods

### 2.1. Study Design

This was a split-mouth randomized case/double-control histological human study. Patients with bilateral edentulous posterior maxilla and requiring implant therapy for fixed prosthetic rehabilitation were eligible for entering this study, provided that they fulfilled the study inclusion criteria (Table 1).

All procedures were performed in accordance with the recommendations of the Declaration of Helsinki for investigations with human subjects [20]. All patients were thoroughly informed about the procedures and signed an informed consent form. The study was approved by the Ethics Committee at the Saint Joseph University of Beirut, Lebanon (USJ-2018-56). Preoperative evaluation included a clinical examination of the edentulous ridges and natural dentition, as well as a cone beam computed tomography (CBCT) of the relevant sector. Patients underwent a prosthodontic evaluation for treatment planning, and all surgeries were performed by the same experienced surgeon (C.M.) at the Oral Surgery Department, Faculty of Dental Medicine, Saint Joseph University (Beirut, Lebanon) between February 2019 and July 2021. Study limitations were mainly the low number of samples, which is normal in this type of research involving human biopsies, but the split-mouth study design and histological analysis conferred more relevance and importance to our results.

### 2.2. Surgical Procedure

First, 10 min prior to surgery, patients were asked to rinse with 0.2% chlorhexidine digluconate solution for 1 min approximately. After local anesthesia, a full-thickness flap was elevated at the crestal level. The implant bed was then prepared following a standard drilling protocol for placement of 4.5/5 × 10 mm implants (AnyRidge, MegaGen, Gyeongbuk, Republic of Korea) in both maxillary posterior regions. Standard drilling for implants 3.5 × 8.5 mm (MegaGen, Gyeongbuk, Republic of Korea) was also performed posteriorly to the prepared sites in both areas. On each side, 2 standard and 2 micro-implants with 2 different surfaces (XPEED**^®^** and SLA) were placed (Figure S1). The XPEED**^®^** surface was characterized by an average roughness value (Ra) of 1.63 ± 0.22 (µm) and a concentration of Ca^2+^ ions of 23.99% (At%). All implants were placed into the osteotomies until final seating. The tested implants were oriented so that the marked line on the external hexagon was parallel to the buccal plate. This line guided the future sectioning plan in a way that BIC was analyzed on the mesial and distal proximal bone instead of the compact buccal or palatal plates. Flap was the approximated and sutured for a submerged healing protocol. Patients were prescribed analgesics and prophylactic antibiotic coverage (amoxicillin 2 g/daily or in case of allergy clindamycin 600 mg/daily) for 7 days and oral rinses of 0.12% chlorhexidine gluconate for 15 days following implant placement. Patients were recalled for suture removal 7 to 10 days following surgery. Tetracycline administration time was randomly performed at 2 or 4 weeks. Following this step, second-stage surgery was performed accordingly at either 4 or 6 weeks to connect healing screws on definitive implants. Then, micro-implants were retrieved with surrounding bone using a 6.5/5.5 mm diameter trephine drill at either 4 weeks (MAR^3w^) (*n* = 6) or 6 weeks (MAR^5w^) (*n* = 11). The retrieved specimens were immersed in 10% neutral buffered formalin solution and sent for histomorphometric evaluation to determine MAR around the implant surface through the analysis of tetracycline-labeled bone.

### 2.3. Working Mechanism and Fluorochrome Use

**Figure 1:**
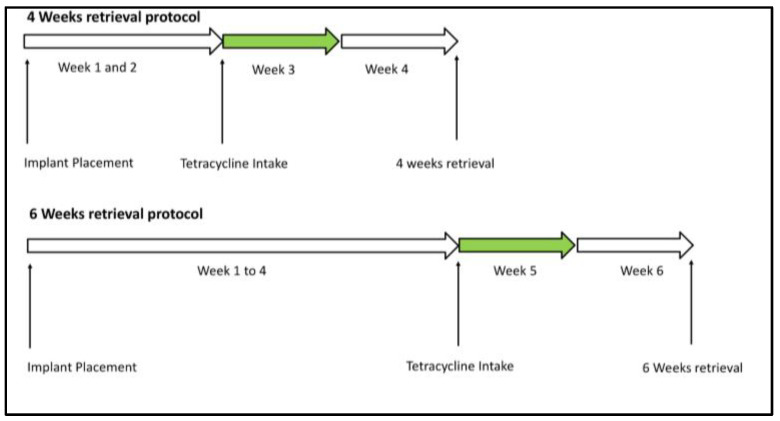
Tetracycline bone labeling protocol: A tetracycline dose was given at either two or four weeks, and labeled bone was observed during the third and fifth weeks.

In fluorescence microscopy, light is transmitted through the sample toward an objective lens, whereas in epifluorescence microscopy, light is transmitted through the objective lens onto the sample. Consequently, epifluorescence microscopy eliminates the need to filter out unwanted light directly from the source. In brief, an excitatory light beam is passed through filters, a specific dichroic mirror, and a standard objective lens, reaching the specimen where the light is partially absorbed and reflected by the specimen itself. The fluorophores in the specimen emit light of a longer wavelength than the light used to excite the fluorophore. The reflected and emitted light can be observed after focusing. With the proper light filters and mirror, only the emitted fluorescent light is allowed to pass through to the eyepiece or detector. Fluorochrome labels, after binding to calcium ions, are incorporated at sites of mineralization in the form of hydroxyapatite crystals Ca_5_(PO_4_)_3_(OH). This means that the label indicates all sites of mineralization in the specimen. In the first 24–36 h after administration, the label is stabilized. As a result, the fluorescent label demarcates the mineralization front at the time of administration and can be detected in histological sections without any further staining or decalcification. Bone formation can be followed in time in relation to fluorochrome administration and drug clearance from the blood. Each patient received 4 administration doses of 500 mg of tetracycline (tetracycline hydrochloride C_22_H_24_N_2_O_3_, HCl molecular weight 480.90 CAS no 64-75-5) every 12 h for 2 days (48 h) starting from 2 weeks prior to biopsy retrieval. Only one course of tetracycline was administered; then, based on the drug half-life (12 h for HCl tetracycline), the time lapse from 0 to less than 0.5 mg blood concentration was calculated (7.1 days) (Table 2). Therefore, biopsies retrieved at 4 weeks (MAR^3w^) allowed for the visualization of labeled bone corresponding to the 3rd week of healing (starting with the 1st administered dose of tetracycline at 2 weeks and spanning 7 days after that as per its blood concentration); those retrieved at 6 weeks (MAR^5w^) corresponded to the 5th week of healing (Figure 1).

The mineral apposition rate (MAR) represents the mean speed at which an osteoid seam is mineralized. This entails the measurement of mean marked bone thickness near implant surfaces, corrected for obliquity, measuring two independent slides obtained from each retrieved sample at different magnifications: ×10, ×20, ×40. A total of 15 measurements for each specimen were collected. Collected data were divided by the number of hours (in days) necessary to reach the theoretical 0.5 mg tetracycline blood concentration, based on the quantity of administered drug and its half-life. The following equation of bone apposition was used to calculate *MAR* (μm/day) (1):

(1) MARμm/day=∑i=0n e π4n t

where $\sum_{i=0}^{n}.$ is the sum of all the measurements of labels’ width, *e* is the micrometer calibration factor (mm), *π*/4 is the obliquity correction factor, *n* is the total number of measurements, and t is the time interval expressed (days).

### 2.4. Histological Processing

The retrieved biopsies were carefully rinsed with a cold 5% glucose solution to remove blood residuals, maintaining the correct osmolarity (278 mOsm/L); then, they were placed in a sealed container containing 10% phosphate-buffered formalin solution at pH 7.1. Specimens with labeled bone were packed with aluminum foil to ensure minimal exposure to light as fluorochromes are light-sensitive. The specimens remained in the formalin solution for one and two weeks depending on size. After the fixation process, the specimens were placed in ascending concentrations of ethanol, shielded from light, and under constant agitation at 70% ethanol for 1 week, 80% ethanol for 1 week, 90% ethanol for 1 week, and 100% ethanol for 1 week. After dehydration, the specimens were pre-infiltrated for ten days in a 50% resin/alcohol solution (LR White, London Resin Co., Ltd., Aldermaston, UK) and then embedded in resin (Technovit 7200 VLC, Kulzer, Wehrheim, Germany). After polymerization, undecalcified cut sections, 50 μm each, were prepared, ground, and polished by using the TT System (TMA2, Grottammare, Italy). For fluorochrome analysis, specimens were left unstained and larger in thickness, since thicker sections produce brighter fluorescence. The investigation was performed by the same experienced researcher (T.T.) by means of a Leica DM 2000 fluorescent microscope (Leica Microsystems, Wetzlar, Germany), which was connected to a high-resolution digital camera ICC50HD (Leica Microsystems, Wetzlar, Germany). A histometric software package with image-capturing capabilities (Image-Pro Plus 6.0, Media Cybernetics Inc., Bethesda, MD, USA) was used. To ensure accuracy, the software was calibrated for each experimental image using ‘Calibration Wizard’, a feature that reports the number of pixels between two selected points: implant length (8.5 mm). The linear remapping of the pixel numbers in microns was used to calibrate the distance. MAR measurement was performed by measuring the marked bone (labeled) linear width near different implant surfaces. For each calibrated image, calculations were performed based on the distance between the start and the end of the labeled bone rim over time (2 weeks of administration plus 7.1 days) at the interface region using Equation (1). Tetracycline light excitation was 390 (nm), while the emission light wavelength was 560 (nm) as in the literature [21,22].

### 2.5. Statistical Analysis 

Descriptive statistic was obtained for SLA and XPEED**^®^** MAR test data. The results were statistically inferred using the unpaired Student’s *t*-test for MAR test data for both MAR^3w^ and MAR^5w^. The level of statistical significance was set at *p* < 0.05. Polynomial linear regression (f = y0 + a*x). Statistical analyses were performed using IBM SPSS Statistics v. 3.5 (IBM Corp., Armonk, NY, USA).

## 3. Results

### 3.1. Clinical Outcome

All implants (SLA and XPEED^®^) healed uneventfully, as did all biopsy sites, and no complications were noted during the whole course of this study. At 1 year follow-up, all sites showed uneventful healing with no clinical or radiographic complications.

### 3.2. Histomorphometric

**Figure 2:**
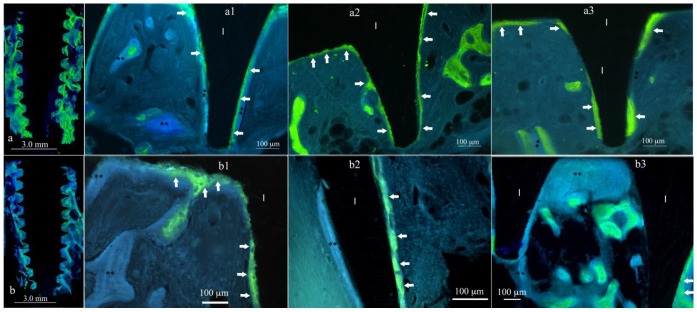
XPEED MAR^3w^ vs. SLA MAR^3w^. Image reconstruction of XPEED MAR^3w^ in (**a**) and of SLA MAR^3w^ in (**b**). Marked bone appears in green. The white arrows indicate the position of the newly formed bone under mineralization that appeared to be mainly attached to the implant surface (I) of the XPEED implants than that of SLA implants. XPEED^®^ implant surface demonstrated active and early osteoinductive and osteoconductive properties associated with an increased osteoblast activity, while the SLA implant surface demonstrated less osteoinductive and osteoconductive properties associated with increased osteoblast activity; (**) shows bone deposition starting from week 3 until retrieval time (4th week). The images (**a1**,**a2**,**a3**) belong to the same group (XPEED) but to different patients, as are images (**b1**,**b2**,**b3**). At the same time, (**a1**,**b1**), (**a2**,**b2**), and (**a3**,**b3**) belong to the same patient.

The mineral apposition rate (MAR, μm/day) was the amount of bone accretion labeled during the 160 h of tetracycline administration, considering the drug half-life (Table 2) that occurred in each individual time point. The measure was related to the maxillary tuberosity bone (implant site insertion area) during bone healing (Table 3). After three weeks of healing, the MAR^3w^ value was 2.0 (±0.18) μm/day for XPEED*^®^* implants and 1.5 (±0.10) μm/day for SLA implants. The difference was statistically significant (*p* = 0.017) (Table 4). In both types of implant surfaces, at this early time point (3 weeks), newly formed bone appeared to grow directly in contact with the implant surface (Figure 2), with significantly higher values for XPEED*^®^* specimens when compared to SLA. In any case, after 3 weeks of healing time for each implant surface, it was possible to observe direct osseointegration. This was related to the osteoconductive activity occurring at the implant surfaces. At the same time, an osteoinductive action was noted as increased osteoblast activity demonstrated by the higher MAR values.

**Figure 3:**
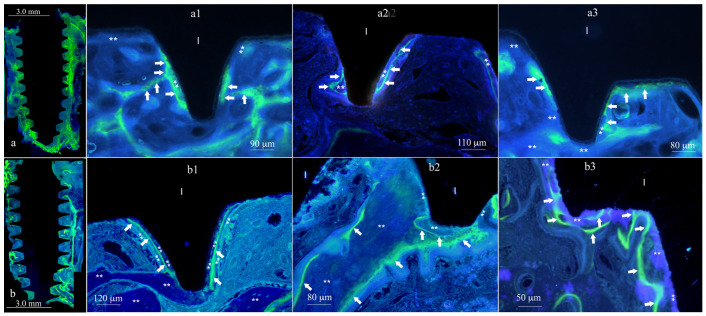
XPEED MAR^5w^ vs. SLA MAR^5w^. Image reconstruction of XPEED MAR^5w^ in (**a**) and of SLA MAR^5w^ in (**b**). Marked bone appears in green. The white arrows are inside the newly formed bone near the implant surface (I). Retrieval time was 6 weeks, with (**) indicating bone deposition having occurred the first 4 weeks and during the 6th week of healing. Marked bone appears to be thinner than that obtained in XPEED MAR^3w^ specimens, implying a slightly decreased osteoblasts activity. The images (**a1**,**a2**,**a3**) belong to the same group (XPEED) but to different patients, as are images (**b1**,**b2**,**b3**). At the same time (**a1**,**b1**), (**a2**,**b2**), and (**a3**,**b3**) belong to the same patient.

**Figure 4:**
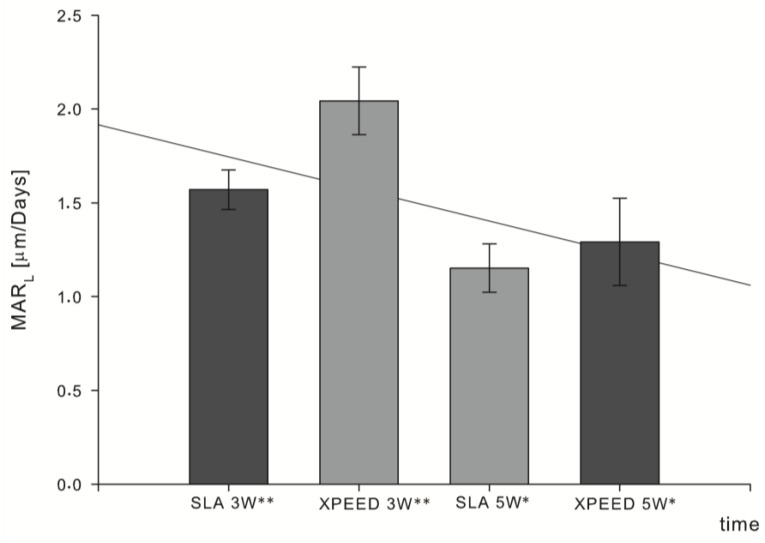
The mineral apposition rate, expressed as microns of bone mineralized for each day with respect to the implant surfaces at 3 and 5 weeks. The coefficient of determination of the linear regression analysis (R^2^ = 0.284) showed decreased osteoblast activity (*p* < 0.005) for MAR^5w^ weeks compared to MAR^3w^. Moreover, this trend appears to be associated with a decrease in the difference between the two tested implant surfaces; ** statistically significant different *p* = 0.017; * statistically significant different *p* = 0.046.

After five weeks, the MAR^5w^ values were 1.2 (±0.10) μm/day for XPEED*^®^* implants and 1.1 (±0.10) μm/day for SLA. Newly formed bone appeared to grow directly in contact with already deposited bone, with a decreased activity in direct contact with the implant surface (Figure 3). Compared to SLA implants, XPEED*^®^* implants appeared to retain a statistically significant difference (*p* = 0.046) (Table 4). XPEED MAR^3w^ values were 25% higher than those of the SLA MAR^3w^ control group. While both MAR^5w^ groups (test and control) showed significantly lower values compared to MAR^3w^, the XPEED MAR^5w^ group still showed higher values than SLA MAR^5w^. The overall evaluation by linear regression analysis for both time vs. implant surfaces showed a decrease in the osteoblast activity for MAR^5w^ compared to MAR^3w^ (*p* < 0.005) (Figure 4). MAR is an important histomorphometric variable for the calculation of dynamic implant bone osseointegration (DIBO) that is a reliable index of osteoblast function or osseointegration process and represents an important clinical indicator for implant loading administration.

## 4. Discussion

Implantology has come a long way since Branemark’s machined surface implant was developed almost 60 years ago, with the raw material type (titanium) being almost the only common factor between the modern-day fixture and its ancestor. Variables such as implant stability, placement, and loading protocols, as well as overall success rates, have all been extensively enhanced, and the future seems to hold even more progress. Osseointegration around dental implants has been extensively studied, and several critical factors were identified as variables such as bone turnover, implant bed preparation, implant design, and surface treatment, leading to a large degree of heterogeneity in bone response [23,24].

Implant surface modification was one of the key elements undergoing this considerable improvement since modern implant surfaces allow for faster, better, and safer osseointegration [25]. During implant bed preparation, local bleeding subsequent to drilling is considered the first step of bone healing [26]. After implant insertion, implant mechanical fixation into bone is defined as primary stability [6]. Secondary stability starts after new bone apposition takes place [23]. At this point, the type of engagement between the implant and bone shifts from a mechanical to a biological bond [27]. The overall implant stability consists of primary stability, which decreases over time, and secondary stability, which increases with time [23]. From a clinical point of view, the overall stability and the time by which it is reached are very important. The gold standard generally used to investigate the amount of bone in contact with an implant surface is histomorphometry, which is expressed as the bone-to-implant contact percentage (BIC) [10]. However, numerous publications concerning BIC do not specify which bone in contact with the implant is measured (new, old, or total bone) [28,29], knowing that this aspect plays an important role if we consider the early healing phases. This last aspect is not well investigated, and no data were present about how faster osseointegration is important for implants placed under immediate loading conditions. It has already been reported that nanostructured calcium-incorporated surface topography can also modulate cellular behavior, and studies have shown that nanoscale structural features can positively influence cell adhesion and subsequent hMSC proliferation and differentiation [30,31,32]. He et al., in an in vivo animal study, showed that early bone formation around an implant is significantly accelerated by nanoporous surfaces, through the modulation of cell behavior and osteogenesis [33]. Mangano et al. compared 8-week BIC on 24 implants (12 XPEED**^®^**, 12 machined), and the histomorphometric evaluation revealed a clear superiority of the tested implants compared to control, with 39.7 (±8.7)% and 21.2 (±4.9)%, respectively, a statistically significant difference in similar bone conditions [34]. Also, the histomorphometric analysis of XPEED**^®^** specimens retrieved from human subjects showed significantly higher BIC when compared to SLA [10]. In a recent publication, nanostructured calcium-incorporated surface (XPEED**^®^**) was shown to significantly enhance bone deposition around implants at early healing stages when compared to both SLA and turned surfaces [10]. This was accomplished by the histological measurement of bone-to-implant contact in human biopsies at 4- and 6-week healing intervals. The present study was conducted using the same protocol; additionally, patients were prescribed tetracyclines 2 weeks prior to biopsy retrieval in order to measure MAR with fluorochrome bone labeling.

The fluorescence of tetracycline antibiotics in bone was first described in the literature in the late 1950s [35]. These molecules were shown to interact with calcium and the organic matrix of newly proliferated bone, specifically at sites of new bone formation [36]. Frost et al. pioneered the clinical use of tetracycline as a fluorochrome marker for bone [37]. Interestingly, these investigations indicate that tetracycline antibiotics can have divergent dose-dependent effects on osteoblastogenesis. Duewelhenke et al. found that incubating primary human osteoblasts with tetracycline (60–80 μg/mL) inhibited cell proliferation by 20% [38]. Gomes et al. investigated the effect of doxycycline (1–25 μg/mL) and minocycline (1–50 μg/mL) on the proliferation, differentiation, and function of human bone marrow-derived osteoblastic cells in culture [39]. A 1 μg/mL dose of doxycycline or minocycline increased the proliferation of osteoblastic cells without altering their functional activity. Rauch et al. reported that the dynamic bone formation data can only be measured in vivo when patients have received a tetracycline label prior to biopsies [40]. In an in vitro study, it was shown that tetracycline labeling may be used for topographic and quantitative information on bone mass elaborated on a calcium phosphate substrate [41]. In vivo tetracycline deposition in bone formation sites facilitated the studies of undecalcified sections by fluorescence microscopy, making them a safe time marker for the direct microscopic measurement of bone formation dynamics [42,43]. Since then, tetracyclines have been considered a very valuable tool in the estimation of MAR as well as the localization of bone formation sites [11].

This is very useful in oral implantology, where an objective histological assessment of bone behavior around dental implants is crucial in the advancement of implant surface performance [18].

In a study on implants placed in mongrel dogs’ upper and lower jaws, the animals were administered tetracyclines 5 to 10 days prior to biopsy retrieval, at 15, 30, 60, and 120 days after implant placement, and the authors were able to use tetracycline labeling to detect active bone growth at implant interface [44].

In a comparative study comparing the effect of two implant drill cooling methods on bone structure, polyfluorochrome sequential labeling was used to highlight the superior quality and speed of new bone formation in the metaphyseal spongy bone relative to diaphyseal compact bone and to determine the time of bone formation onset as well as resorption processes [45].

In a human study comparing bone remodeling around loaded and unloaded implants retrieved 6 months after placement, patients were administered tetracycline 30 and 60 days prior to retrieval, and the distance between the two resulting fluorescent lines was measured on biopsies in order to calculate the bone remodeling rate [46]. This protocol also allowed for the calculation of bone labeling percentage for an objective assessment of new bone formation.

Another study used tetracycline labeling along with other histological and radiographic methods for a comprehensive implant surface performance evaluation in a rabbit model at early healing stages [19], and according to the authors, these methods allowed for an objective histomorphological, osteogenic, mineralization, and integrative assessment of bone dynamics and mineral apposition rate around the implant surface.

However, and to the authors’ knowledge, there has not been a publication to date measuring early-stage MAR on implants placed in human subjects. All implants in this study healed uneventfully regardless of the administered tetracycline dose, and no adverse effects were noted in any of the patients, suggesting that this labeling protocol is safe and does not interfere with surgery outcome. None of the patients complained of any clinical side effects related to tetracycline intake, especially given the fact that the dose was minimal, and tetracycline has been a widely and safely used medication for many decades.

Our findings showed that newly formed bone appeared in direct contact with both types of tested surfaces in MAR^3w^ biopsies. MAR values were, respectively, 2.0 (±0.18) μm/day for XPEED and 1.5 (±0.10) μm/day for SLA, showing a statistically significant difference (*p* = 0.017). The null hypothesis under study was therefore rejected. MAR represents the mean speed at which bone mineral is deposited, and in this study, it allowed for the comparative assessment of two types of implant surfaces, as well as the establishment of a certain timeline of bone formation at early healing stages.

Our results showed that, for both MAR^5w^ groups, osteoblastic cell activity decreased by approximately 50% compared to MAR^3w^ when we consider the change in MAR values for both groups. When comparing MAR^3w^ to MAR^5w^ results, the mineralization front (μm/day) varied from 2.0 (±0.18) to 1.2 (±0.10) for XPEED groups and from 1.5 (±0.10) to 1.1 (±0.10) for SLA groups, respectively. In a split-mouth study on SLA and SLActive**^®^** implants placed in human mandibles and retrieved at either 7, 14, 28, or 42 days, it was shown that the superior hydrophilic surface yielded higher BIC results at 14 and 28 days but had comparable performance to the untreated surface at 7 and 42 days, suggesting that enhanced surface performance will result in faster but not necessarily better osseointegration [47]. However, more importantly, it highlights a certain decrease in the speed of bone deposition on implant surfaces between 4 and 7 weeks, which is in line with the current study’s decreased MAR^5w^ values. This does not suggest any unfavorable effect on osseointegration outcome and only helps draw certain associations between surface types and loading protocols. That being said, superior histological values are of little interest to both clinicians and patients if they have no clinical significance since what matters most is the actual implant behavior when placed in functional clinical conditions; in order to meet current standards of care as well as patient expectations, the faster the treatment the better. As a matter of fact, in a previous clinical study by the authors on 40 implants with Ca-incorporated nanostructured surface (XPEED**^®^**), the early monitoring of implant stability allowed for the application of a 4-week loading protocol with a 100% success rate [6]. This was justified following the weekly monitoring of ISQ values, which showed little to no drop during the first 4 weeks and remained >70. McCullough et al. reported similar findings on the same type of surface and stated that the XPEED**^®^** surface, coupled with knife-edge threads, may result in significantly reduced bone remodeling around implants and the quasi-absence of ISQ 3-week drop [48]. This allowed the authors to link histological and experimental findings to clinical reality and further ascertain that this type of surface allows for accelerated treatment modalities. Aligning these findings with the present ones further supports the superior performance of calcium-incorporated SLA surfaces in promoting bone formation around implants at early healing stages. These results indicate that the more complex nanotextured implant surfaces (XPEED**^®^**) demonstrated excellent biocompatibility and bioactivity in terms of osteoconductivity and bone-bonding potential, leading to a more efficient osteogenic process in the first month of healing. This enhanced performance in the posterior maxilla has already been described for XPEED**^®^** surfaces in a clinical study by the authors, reporting sufficient stability and insignificant drop in RFA values at 4 weeks, facilitating implant loading in low-quality bone, even after this short healing period (6). Although the study design with the split-mouth method and histological analysis confers great relevance and importance to our results, some study limitations emerge, mainly related to a monocentric study and the low number of samples which, perhaps, is plausible in research involving human subjects. Therefore, further investigations with more relevant sample sizes are needed in order to establish the timeline of the bone healing, early after implant placement.

## 5. Conclusions

Nanostructured calcium-incorporated surfaces demonstrated superior biocompatibility and bioactivity compared to traditional surfaces; they were found to significantly promote early bone formation around implants. Tetracycline labeling proved to be a safe and effective method for evaluating bone dynamics around implants, offering valuable and objective insight into implant surface performance in clinical conditions. When comparing XPEED^®^ and SLA surfaces, histological assessment with tetracycline labeling demonstrated higher MAR values during early healing stages as bone formation seemed to occur faster around implants with XPEED^®^ surfaces. Three-week MAR values showed higher bone formation when compared to five-week samples in both control and test specimens. The clinical relevance of the present study supports the use of nanostructured calcium-incorporated surfaces in cases requiring shortened healing protocols and accelerated treatment modalities, even in low-quality bone.

## Figures and Tables

**Table 1:** Criteria for patient selection [10].

Inclusion Criteria	Exclusion Criteria
Height of the residual bone crest in the programmed implant site ≥ 9 mm and thickness ≥ 7 mm.	Myocardial infarction within the past 6 months
Availability, in each sector, of sufficient mesiodistal space allowing for the placement of 2 standard-sized implants and at least 2 mini-implants (3.5 × 8.5 mm) for retrieval	Poorly controlled diabetes (HBA1c > 7.5%)
Healed bone crest (≥3 months elapsed after extraction or tooth loss).	Coagulation disorders
Age > 18 years	Radiotherapy to the head/neck area within the past two years
Ability to examine and fully understand the study protocol	Present or past treatment with intravenous bisphosphonates
	Immunocompromised patients
	Psychological or psychiatric problems
	Alcohol or drug abuse
	Poor oral hygiene and motivation (full mouth plaque score > 30% and/or full mouth bleeding score > 20%)
	Uncontrolled periodontal disease

**Table 2:** Summary of tetracycline administration and drug half-life.

Dose Taken	Dose at Half-Life Time (12 h)	Time Lapse (hours)
500 mg	250 mg	12
500 mg	325 mg	24
500 mg	412.5 mg	36
500 mg	456.25 mg	48
-	228 mg	52
-	114 mg	64
-	57 mg	76
-	28.5 mg	88
-	14.25 mg	100
-	7.12 mg	112
-	3.56 mg	124
-	1.78 mg	136
-	0.89 mg	148
-	0.445 mg	160

**Table 3:** Descriptive statistics.

Column	Size	Missing	Mean	Std Dev	Std. Error	C.I. of Mean
SLA MAR^3w^	3	0	1.570	0.106	0.0611	0.263
XPEED MAR^3w^	3	0	2.043	0.180	0.104	0.447
SLA MAR^5w^	5	0	1.152	0.129	0.0576	0.160
XPEED MAR^5w^	6	0	1.292	0.233	0.0951	0.244
**Column**	**Range**	**Max**	**Min**	**Median**	**25%**	**75%**
SLA MAR^3w^	0.200	1.690	1.490	1.530	1.500	1.650
XPEED MAR^3w^	0.330	2.250	1.920	1.960	1.930	2.178
SLA MAR^5w^	0.310	1.280	0.970	1.140	1.068	1.273
XPEED MAR^5w^	0.670	1.650	0.980	1.280	1.130	1.430
**Column**	**Skewness**	**Kurtosis**	**K-S Dist.**	**K-S Prob.**	**Sum**	**Sum of Squares**
SLA MAR^3w^	1.458	--	0.314	0.268	4.710	7.417
XPEED MAR^3w^	1.636	--	0.345	0.175	6.130	12.590
SLA MAR^5w^	−0.475	−0.867	0.220	0.499	5.760	6.702
XPEED MAR^5w^	0.336	0.164	0.170	0.690	7.750	10.282

**Table 4:** The results of unpaired Student’s *t*-test.

Group Name	N	Missing	Mean	Std Dev	SEM
SLA MAR^3w^	3	0	1.570	0.106	0.0612
XPEED MAR^3w^	3	0	2.043	0.180	0.104
		Difference −0.473			
t = −3.922 with 4 degrees of freedom. (*p* = 0.017)					
95 percent confidence interval for difference of means: −0.808 to −0.138					
The difference in mean values of the two groups is greater than the values expected by chance; there is a statistically significant difference between the input groups (*p* = 0.017).					
**Group Name**	**N**	**Missing**	**Mean**	**Std Dev**	**SEM**
SLA MAR^5w^	5	0	1.150	0.1000	0.0447
XPEED MAR^5w^	6	0	1.290	0.1000	0.0408
		Difference −0.140			
t = −2.312 with 9 degrees of freedom. (*p* = 0.046)					
95 percent confidence interval for difference of means: −0.277 to −0.00302					
The difference in the mean values of the two groups is greater than the values expected by chance; there is a statistically significant difference between the input groups (*p* = 0.046).					

## Data Availability

The raw data supporting the conclusions of this article will be made available by the authors on request.

## Supplementary Materials

The following supporting information can be downloaded at: https://www.mdpi.com/article/10.3390/ma17133341/s1, Figure S1: SEM images at 50.00 K× magnification of implant surfaces SLA (a) and XPEED (b). Arrows in (b) show the CaTiO_3_ structures not present in (a).
