# Supplementary material for: The Mineral Apposition Rate on Implants with Either a Sandblasted Acid-Etched Implant Surface (SLA) or a Nanostructured Calcium-Incorporated Surface (XPEED®): A Histological Split-Mouth, Randomized Case/Control Human Study
Source: Materials (Basel). 2024 Jul 5;17(13):3341. doi: 10.3390/ma17133341 (PMC11243467; doi:10.3390/ma17133341)
Supplement: Supplementary file 1 [file materials-17-03341-s001.zip › materials-3040300-supplementary.pdf]

Supplementary Materials

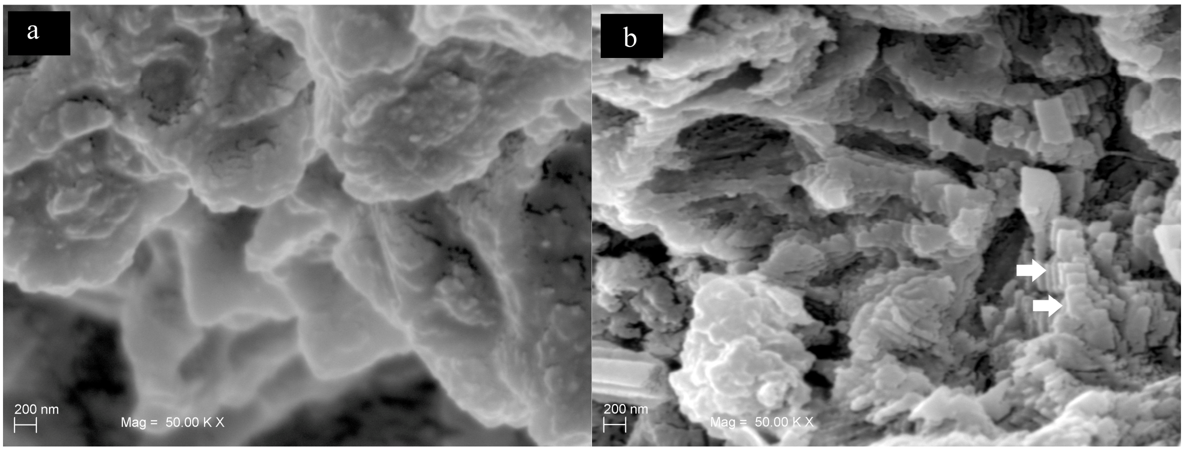

Figure S1: SEM images at 50.00 K× magnification of implant surfaces SLA (a) and XPEED (b). Arrows in (b) show the $CaTiO_3$ structures not present in (a).
